# A Postpartum Case of Non-ST Elevation Myocardial Infarction Due to Coronary Artery Aneurysm After Preeclampsia

**DOI:** 10.7759/cureus.88185

**Published:** 2025-07-17

**Authors:** Yousuf A Almadhoun, Deekshitha Tella, Manasa Dittakavi, Sereen Khourshid, Talal Al-Khawlani

**Affiliations:** 1 Osteopathic Medicine, West Virginia School of Osteopathic Medicine, Lewisburg, USA; 2 Osteopathic Medicine, Charleston Area Medical Center, Charleston, USA; 3 Internal Medicine, Charleston Area Medical Center, Charleston, USA

**Keywords:** acute coronary syndrome, left main coronary artery aneurysm, non-st segment elevation myocardial infarction (nstemi), postpartum, preeclampsia

## Abstract

Acute coronary syndrome (ACS) in the postpartum period is a rare but serious complication, particularly in patients with a recent history of preeclampsia. Preeclampsia is a multifactorial disorder characterized by hypertension, proteinuria, and peripheral edema due to abnormal placentation and vascular remodeling. We present the case of a 21-year-old healthy female patient with no history of cardiovascular disease (CVD), one week postpartum after cesarean delivery for severe preeclampsia, who developed a non-ST elevation myocardial infarction (NSTEMI). Cardiac catheterization revealed a left anterior descending (LAD) artery aneurysm with 99% stenosis, necessitating urgent stenting. Despite the rarity, the aneurysm was suspected to be secondary to preeclampsia-related endothelial dysfunction. The patient was managed with dual antiplatelet therapy, heparin, and blood pressure control. After stabilization and discharge, she maintained regular follow-up with her primary care physician to discuss secondary prevention of further cardiovascular adverse events. Postpartum ACS, especially secondary to coronary artery aneurysm (CAA), is exceedingly rare but highlights the importance of cardiovascular monitoring in patients with a history of preeclampsia. This case underscores the need for further research into endothelial dysfunction and thrombogenicity in preeclampsia to improve screening, prevention, and management strategies.

## Introduction

Pregnancy is a complex physiological state that predisposes individuals to various medical complications, necessitating careful monitoring and intervention. Among these complications, preeclampsia is a significant multisystem hypertensive disorder characterized by new-onset hypertension and proteinuria. It affects approximately 3-7% of nulliparous women and 1-3% of multiparous women [1]. The pathophysiology of preeclampsia is multifactorial, involving endothelial dysfunction, placental ischemia, and an exaggerated inflammatory response [2]. The only definitive treatment remains delivery, although postpartum monitoring is essential due to persistent cardiovascular risks [2]. 

Preeclampsia is a significant risk factor for cardiovascular disease (CVD), affecting up to 15% of pregnancies. Women with a history of preeclampsia face a 28.8% increased risk of developing subsequent CVD [3]. Emerging evidence suggests that the cardiovascular effects of preeclampsia may persist beyond pregnancy [4]. Although acute coronary syndrome (ACS) during pregnancy is relatively rare, its incidence has been rising over the past two decades, with peak occurrence between the final month of gestation and the first two weeks postpartum [4,5]. A United States study examining trends in acute myocardial infarction (AMI) in pregnancy found that 53.5% (2,390 cases) occurred in the postpartum period, compared to 20.6% (922 cases) in the antepartum period and 23.7% (1,061 cases) during labor and delivery [6]. Furthermore, the incidence of AMI in pregnancy has increased 5-8 fold in women over 35 years old [6]. Contributing factors to this rise include higher maternal age and higher rates of smoking and obesity in this population. 

In recent years, growing evidence has linked preeclampsia to an increased risk of long-term CVD, including hypertension, stroke, heart failure, and ischemic heart disease [7]. Women with a history of preeclampsia have been found to have 2-4 fold higher risk of developing cardiovascular events later in life [7]. ACS, a broad term encompassing unstable angina, non-ST elevation myocardial infarction (NSTEMI), and ST-elevation myocardial infarction (STEMI), is rare in younger women but is increasingly recognized as a potential complication in the postpartum period. Pregnancy-induced physiological changes, including increased coagulation, endothelial dysfunction, and hemodynamic stress, contribute to an elevated risk of coronary events [8]. In this context, understanding coronary artery abnormalities such as coronary artery aneurysms (CAAs) becomes crucial, as they may represent an underrecognized but serious cardiovascular complication in the postpartum period.

CAAs are rare, with an estimated prevalence of 0.3-5% in patients undergoing coronary angiography. They are defined as localized dilations of the coronary artery exceeding 1.5 times the diameter of the adjacent normal segment [9]. The etiology of CAAs is diverse, including atherosclerosis, connective tissue disorders, vasculitis, Kawasaki disease, and iatrogenic causes. The clinical significance of CAAs varies, with some cases remaining asymptomatic while others present with complications such as thrombosis, embolism, rupture, or myocardial infarction [9]. The mechanisms linking pregnancy-related hypertensive disorders to CAA formation remain poorly understood but may involve endothelial dysfunction, chronic vascular inflammation, and exacerbated arterial remodeling. Furthermore, pregnancy-associated spontaneous coronary artery dissection (SCAD) and CAA have been well documented in postpartum patients, raising concerns about underlying vascular vulnerability in this population [10]. Given the increased thrombotic risk in the peripartum period, CAA in postpartum women poses a unique diagnostic and therapeutic challenge. 

This case report presents a unique instance of ACS secondary to the left anterior descending (LAD) artery aneurysm in a postpartum patient with a history of severe preeclampsia. CAAs are uncommon, particularly in young women, and their association with preeclampsia remains underexplored. By examining this case, we aimed to highlight the potential mechanisms linking preeclampsia to postpartum coronary events, the importance of early recognition, and the need for long-term cardiovascular surveillance in women with a history of hypertensive disorders of pregnancy. 

## Case presentation

A 21-year-old female patient, gravida 2 para 2, with a history of severe preeclampsia, presented to the emergency department (ED) with sudden-onset substernal chest pain radiating to her left arm, associated with diaphoresis and nausea. She had delivered via cesarean section one week prior at 36 weeks of gestation due to severe preeclampsia. Originally, she was induced into labor, but due to recurrent late decelerations, she was taken to the operating room for a cesarean section. She had no prior history of hypertension, diabetes, hyperlipidemia, smoking, or family history of early-onset coronary artery disease.

**Figure 1:**
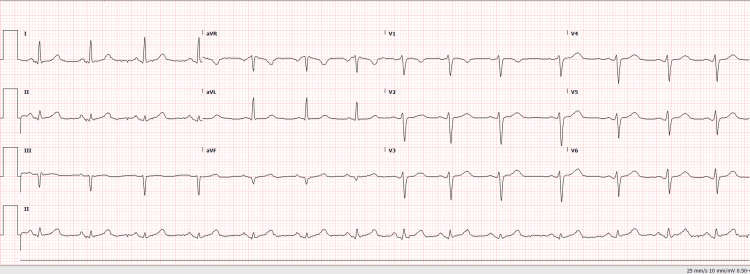
Electrocardiogram (ECG) obtained in the emergency department showing no acute ischemic changes.

On arrival, the patient weighed 60 kg and was hypertensive, with blood pressure readings between 140 and 160 mmHg systolic, a heart rate of 82 bpm, and oxygen saturation of 99% on room air. In the ED, she was placed on a nitroglycerin (NG) drip, and her magnedium was replaced as needed. Her labetalol, which she was taking as an outpatient for preeclampsia, was held in the ED while the NG drip was given. Physical examination was unremarkable, with no signs of heart failure, murmurs, or pericardial friction rub. The initial electrocardiogram (ECG) did not show acute ischemic changes (Figure 1).

Laboratory results showed leukocytosis, elevated erythrocyte sedimentation rate (ESR), elevated absolute neutrophil count, elevated C-reactive protein (CRP), low albumin, slightly low magnesium, and markedly elevated troponins, which prompted the patient to be taken to the catheterization lab despite a normal ECG (Table 1).

**Table 1:** Abnormal lab values obtained in the emergency department.

Test	Result	Reference Range	Interpretation
White cell count	12.2 (High)	3.7 - 11.0 x10³/µL	Elevated (Leukocytosis)
Neutrophil % auto	73.0 (High)	54% - 62%	Elevated
Lymphocyte % auto	19.0 (Low)	25% - 33%	Decreased
Absolute neutrophil	8.90 (High)	1.50 - 7.70 x10³/µL	Elevated
Erythrocyte sedimentation rate (ESR)	53 (High)	0 - 20 mm/hr	Elevated (Inflammation)
C-reactive protein (CRP)	20.1 (High)	<10.0 mg/L	Elevated (Inflammation)
Albumin	3.1 (Low)	3.5 - 5.5 g/dL	Decreased (Hypoalbuminemia)
Magnesium	1.6 (Low)	1.7 - 2.2 mEq/L	Slightly decreased
Sodium, whole blood	135 (Low)	136 - 146 mEq/L	Mild hyponatremia
Troponin I high sensitivity	9637 (Critical)	<15 pg/mL	Severely elevated

**Figure 2:**
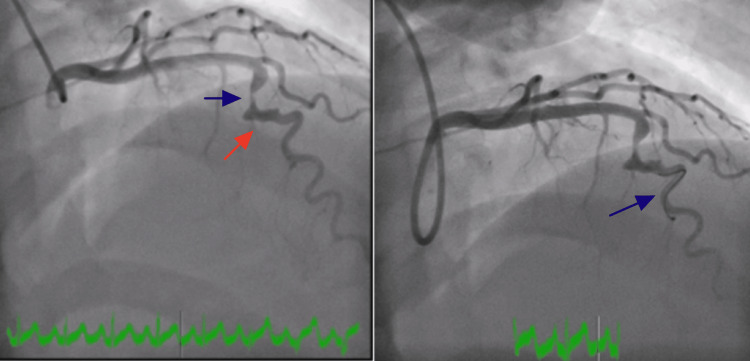
Selective coronary angiography of the left main coronary artery. Severe stenosis of the left anterior descending (LAD) artery is indicated by blue arrows in both panels. The ectatic segment is indicated by a red arrow in the left panel.

**Figure 3:**
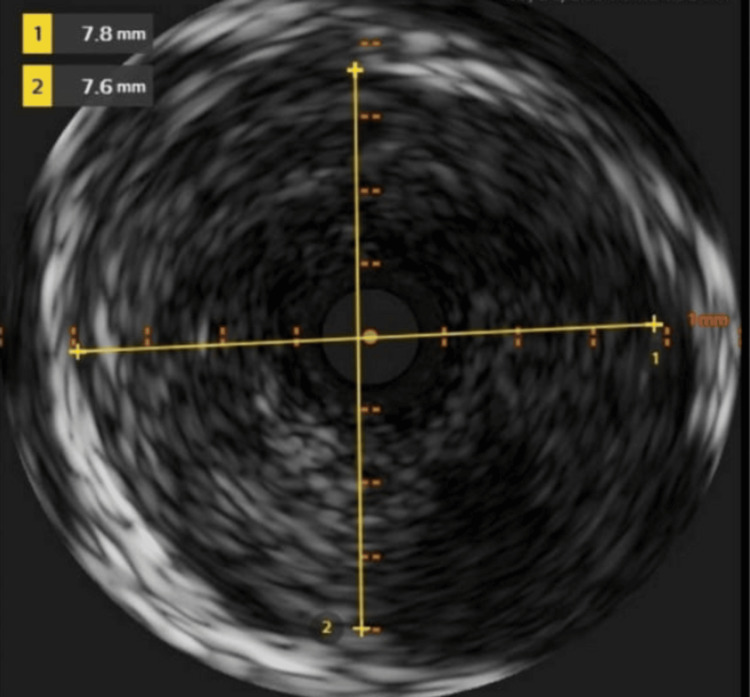
Intravascular ultrasound (IVUS) still frame showing the ectatic segment, with inner luminal diameters measuring 7.8 × 7.6 mm.

**Figure 4:**
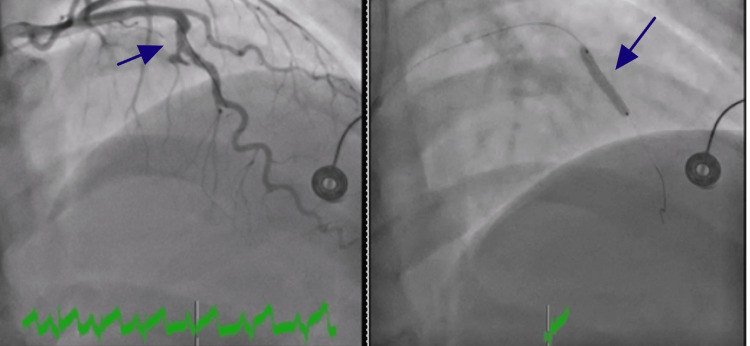
Selective coronary angiography during stent placement across the ectatic segment. The left panel shows positioning of the stent (blue arrow), while the right panel shows successful stent deployment (blue arrow).

Emergent cardiac catheterization revealed an isolated large fusiform aneurysm of the mid-LAD artery. An aneurysmal segment in the mid-LAD was discovered that had 99.9% stenosis with a large clot burden. She underwent successful intervention by placing a 3.5 x 20 mm drug-eluting (SYNERGY) stent (Boston Scientific, United States). Follow-up angiogram showed no evidence of stent edge dissection, vessel perforation, or distal embolization. Images of left heart catheterization and intravascular ultrasound (IVUS) showing a fusiform aneurysm of the mid-LAD are presented in Figures 2-4. IVUS was performed to provide detailed cross-sectional imaging for precise diameter measurements and the extent of the aneurysm.

**Figure 5:**
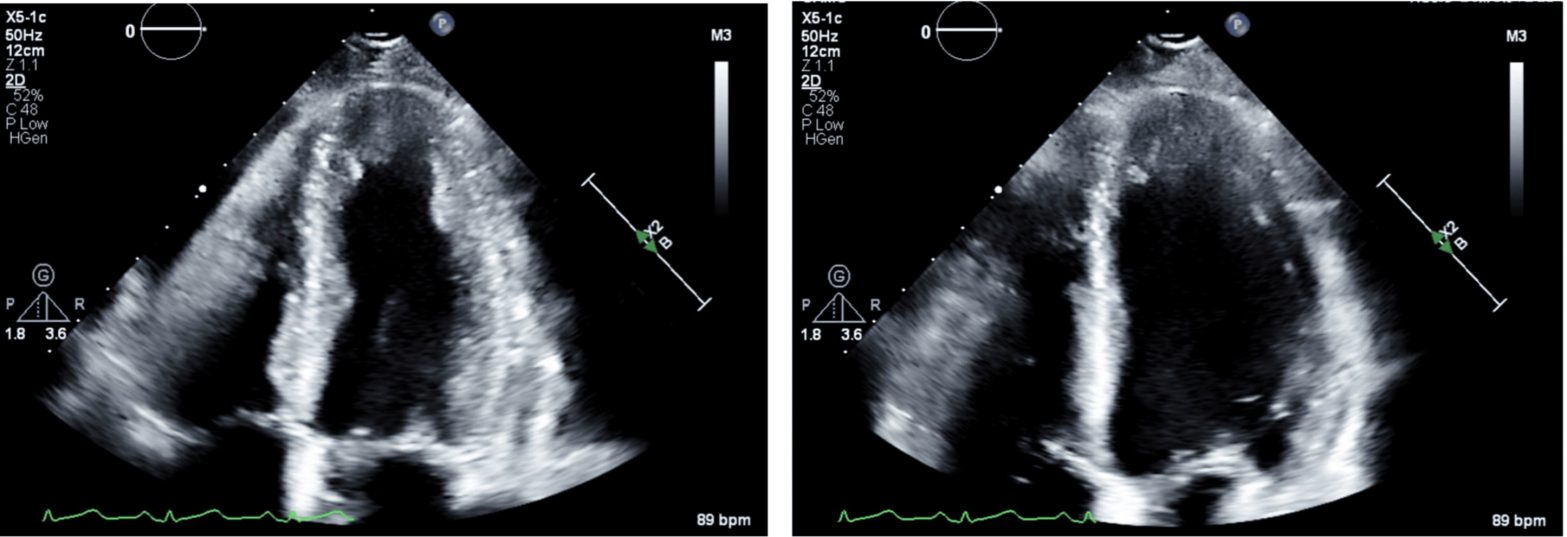
Apical four-chamber echocardiographic views demonstrating akinesis of the apex. The left panel shows the heart during systole, and the right panel during diastole, consistent with a regional wall motion abnormality.

Echocardiography performed post-stent revealed mild left ventricular dysfunction with akinesis of the cardiac apex but preserved overall ejection fraction (Figure 5).

The patient was initiated on dual antiplatelet therapy and anticoagulation (heparin). Given the uncertain long-term prognosis of CAAs in postpartum patients, a multidisciplinary discussion involving cardiology, maternal-fetal medicine, and cardiovascular surgery was conducted. Conservative management with close outpatient follow-up was recommended. The patient was advised to schedule more imaging in coordination with her primary care physician and cardiologist to further understand the risk of CVD, especially if she planned to become pregnant again in the future.

The patient’s recovery was uneventful. She was discharged home on guideline-directed medical therapy (GDMT), including aspirin, ticagrelor, metoprolol, and atorvastatin, with close outpatient follow-up.

This case underscores the potential cardiovascular complications in postpartum women with a history of preeclampsia, highlighting the need for vigilance in recognizing non-atherosclerotic causes of myocardial infarction, particularly CAAs, in this population.

## Discussion

ACS in the postpartum period is a rare but significant clinical entity, often presenting with atypical features that can complicate timely diagnosis. One study estimated an incidence of 1.8 per 100,000 deliveries, with many cases linked to underlying conditions such as preeclampsia or peripartum cardiomyopathy [3]. ACS is typically caused by atherosclerotic plaque rupture, coronary thrombosis, or vasospasm, all of which can lead to myocardial ischemia. AMI is already rare in women under 40, accounting for less than 0.7% of AMI cases [11]. Unique physiological and pathological conditions, such as hypercoagulability and vasospasm, are thought to play a role in the development of ACS in this population. This case is particularly notable given the patient’s history of preeclampsia, which is associated with systemic endothelial dysfunction, hypertension, and an increased inflammatory state.

Moreover, a coronary aneurysm, as seen in this patient, leading to an AMI, is extremely rare. Coronary aneurysms in the postpartum period are typically linked to a sequela of coronary artery dissection, with an estimated incidence of around one in 20,000 to 30,000 births. Yet, this is still the most common complication of an aneurysm. NSTEMI, as documented in this case, is an exceedingly rare sequela of a coronary aneurysm [11].

Preeclampsia significantly elevates the risk of cardiovascular events, even beyond the pregnancy period, due to its lasting impact on vascular health. This risk is particularly elevated in the early postpartum years, suggesting that cardiovascular damage initiated during pregnancy continues to evolve postpartum. Mechanistically, preeclampsia-induced endothelial dysfunction, heightened inflammation, and hypercoagulability contribute to thrombotic events and plaque rupture, which are hallmarks of ACS [12]. The rarity of postpartum ACS is emphasized by its incidence of approximately 2% within 20 years of preeclampsia, compared to 1.2% in those without a history of the condition. However, this risk disproportionately affects women below 50 years of age, amplifying the importance of early cardiovascular risk assessment and intervention in postpartum women with a history of preeclampsia [12,13]. 

The relationship between ACS and preeclampsia remains an area of ongoing research. Pregnancy itself places patients in a procoagulant state, and further investigation is needed to explore endothelial dysfunction commonly seen in preeclampsia, as well as how toxic metabolites released during pregnancy can predispose women to thrombotic events. For example, placental growth factor (PGF) is markedly decreased in preeclampsia compared to normal pregnancy [14]. This hormone is responsible for proper angiogenesis, and low levels can lead to endothelial dysfunction. Additionally, soluble Fms-like tyrosine kinase-1 (sFlt-1) is increased in preeclampsia, which is an antiangiogenic factor that binds and inactivates PGF and vascular endothelial growth factor (VEGF), contributing to a reduction in endothelial repair and abnormal placentation [14]. These pathological processes may explain the sequelae of this patient's presentation. A decrease in PGF and an increase in sFlt-1 can lead susceptible vessels like the coronary arteries to structural and functional damage, predisposing her to a prothrombotic state, infarction, and aneurysm formation. Her low albumin can also contribute to a hypercoagulable state, further exacerbating her clinical and imaging findings. Research related to the connection between preeclampsia and cardiovascular aneurysms is rare. Although this patient’s aneurysm was located in the LAD rather than the aorta, studies have demonstrated that young women with a history of preeclampsia exhibit increased average aortic diameters compared to those without [15]. This observation underscores a potential predisposition to vascular dilation and aneurysm formation in this population, warranting further research into the underlying mechanisms linking preeclampsia to widespread vascular remodeling.

A comprehensive assessment of family history and modifiable risk factors is essential in managing ACS and coronary aneurysms in postpartum women. Despite the rarity of these conditions, pregnancy is inherently a high-risk state due to continuous hormonal and physiological changes. Vigilance for the worst possible outcomes of preeclampsia and ACS can help facilitate early diagnosis, ultimately preventing recurrence and improving outcomes in future pregnancies. Limitations to this case analysis include a lack of data from long-term follow-up to assess possible sequelae of stenting and aneurysm formation. In addition, while she did not have known cardiovascular risk factors, a comprehensive cardiovascular assessment, including imaging and lab tests, was not performed on this patient, potentially missing asymptomatic disease she may have had before her hospitalization.

## Conclusions

Preeclampsia is associated with significant cardiovascular risks during the postpartum period, with ACS due to coronary aneurysm being a rare but serious complication. This case underscores the importance of recognizing postpartum ACS as a potential complication of preeclampsia and highlights the need for early cardiovascular risk assessment in affected women. A multidisciplinary healthcare team is essential for establishing appropriate imaging when suspected risk factors arise, and appropriate clinical follow-ups, even for low-risk patients, can help reduce the likelihood of adverse events from undiagnosed ACS in pregnant and postpartum women. In addition, primary and secondary prevention techniques can be employed through peripartum health counseling and risk modifications for women with a prior history of ACS or preeclampsia.

Given the lasting vascular effects of preeclampsia, more research is needed to elucidate the underlying mechanisms correlating preeclampsia to ACS and aneurysm formation, as well as to better identify effective screening tools for at-risk patients. Improved early detection and targeted interventions can help reduce long-term cardiovascular complications and enhance the quality of life for women affected by preeclampsia in the postpartum period.
